# Harnessing nc886 to overcome immune and apoptotic barriers in adenoviral gene therapy

**DOI:** 10.1016/j.omtn.2025.102493

**Published:** 2025-03-06

**Authors:** Mohamed Hammad, Hossam M. Ashour

**Affiliations:** 1 Developmental and Stem Cell Biology, City of Hope Comprehensive Cancer Center, Duarte, CA 91010, USA; 2 Department of Integrative Biology, College of Arts and Sciences, University of South Florida, St. Petersburg, FL, USA

## Main text

The recent *Molecular Therapy Nucleic Acids* issue features an innovative study by Saruuldalai et al.^1^ This study investigates the potential of an adenoviral vector expressing nc886, a non-coding RNA (ncRNA) with anti-interferon and anti-apoptotic properties, to improve the efficacy of gene therapy. This work addresses two major barriers that have long limited the success of adenoviral vectors in clinical applications: immune activation^2^^,^^3^ and apoptosis.^4^ By leveraging the unique properties of nc886, the study explores a promising strategy to enhance adenoviral vector persistence and therapeutic efficacy, potentially transforming the field of gene delivery.

The authors focus on nc886, an endogenous ncRNA known for its role in regulating the double-stranded protein kinase RNA-activated (PKR) pathway. PKR is a critical mediator of the cellular antiviral response and a driver of apoptosis. When activated, PKR triggers a cascade that includes the upregulation of interferon-stimulated genes (ISGs) and initiation of programmed cell death. These responses are particularly problematic for adenoviral vectors, as they lead to rapid clearance of the vector and reduced transgene expression.

**Figure 1:**
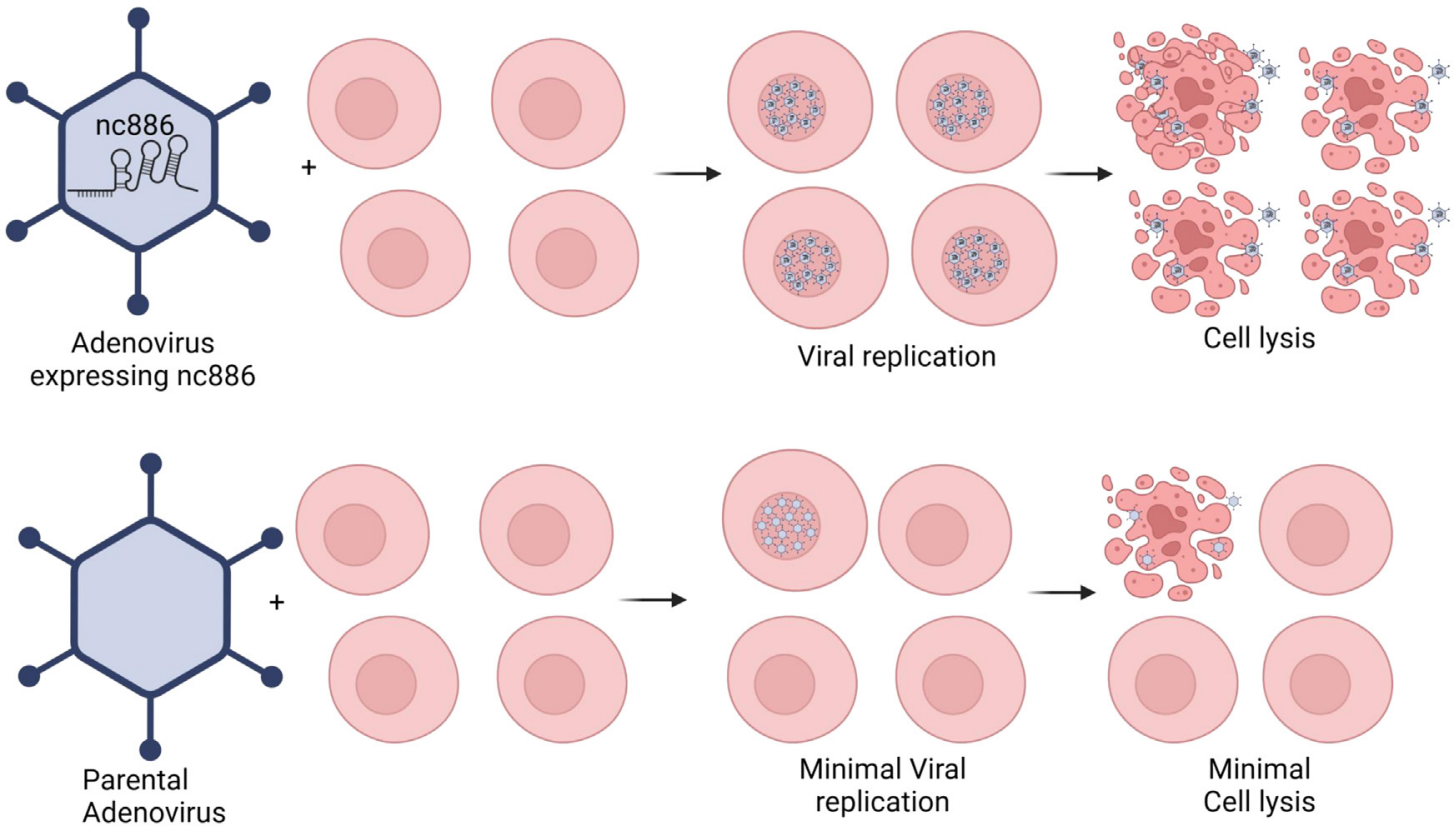
A schematic illustration showing the improved adenoviral vector performance after being engineered to express nc886

The study shows that the authors have developed an rAdV expressing nc886 (AdV:nc886) to investigate whether AdV:nc886 can address the previously mentioned limitations of conventional rAdV vectors. nc886 inhibits PKR activation, effectively suppressing both interferon responses and apoptotic pathways (Figure 1). This dual functionality provides a significant advantage in overcoming two of the most common limitations of adenoviral vectors. The authors’ data reveal that adenoviruses expressing nc886 exhibited reduced immune activation, prolonged transgene expression, and lower levels of apoptosis in both *in vitro* and *in vivo* models. These findings not only validate nc886 as a key regulatory molecule but also highlight its potential to enhance the therapeutic efficacy and applications of adenoviral vectors.

The implications of this study extend beyond the immediate context of adenoviral vectors. By integrating nc886 into their design, the authors have created a platform that could be adapted to various therapeutic areas. In oncology, adenoviral vectors are commonly used in oncolytic virotherapy, where immune clearance and tumor-specific cell death are major concerns.^5^ The nc886-modified vector could improve the persistence and efficacy of oncolytic viruses, enhancing their ability to target and destroy tumor cells. For genetic disorders, gene therapy for inherited diseases often relies on adenoviral vectors for delivery.^6^ The incorporation of nc886 could improve the longevity of gene expression, reducing the need for repeated administrations and minimizing side effects. In vaccines, immune activation can be both a benefit and a drawback in vaccine development. The ability to modulate interferon responses using nc886 may allow for fine-tuning of vaccine efficacy and safety profiles.

The authors make a compelling case for nc886 as a versatile tool in gene therapy. One of the most significant strengths of the study is its focus on both mechanistic understanding and therapeutic application. By elucidating the role of nc886 in suppressing PKR activation, the authors provide a solid foundation for its integration into adenoviral vector platforms.

Moreover, the study employs rigorous experimental methodologies, including both *in vitro* and *in vivo* models, to validate the efficacy of the nc886-modified adenovirus. The use of multiple models strengthens the reliability of the findings and demonstrates the broad applicability of the approach.

While the findings are promising, the study also highlights several challenges that must be addressed before this approach can be translated into clinical practice. One concern is long-term safety, as the modulation of interferon and apoptotic pathways raises questions about potential long-term effects. Prolonged suppression of these pathways could impact host immunity, making individuals more susceptible to infections or malignancies.^7^ Another major challenge is delivery efficiency, as effective delivery of adenoviral vectors to target tissues remains a significant obstacle, particularly for systemic applications. The development of tissue-specific delivery systems will be crucial for translating these findings into practical therapies.^8^

Additionally, off-target effects must be considered. While nc886 appears to enhance vector performance without major side effects in preclinical models, further studies are necessary to ensure that its expression does not disrupt other cellular pathways or lead to unintended consequences. Lastly, variability in the host response presents another challenge, as individual differences in nc886 expression and PKR pathway activity could impact the efficacy of the modified vector.^9^ Personalized approaches may be necessary to optimize outcomes for different patient populations.

This study represents a significant advancement in the field of gene therapy. By incorporating nc886, the authors have introduced a novel strategy to overcome two of the most persistent barriers to adenoviral vector efficacy. The findings pave the way for the development of more efficient and less immunogenic gene delivery platforms. In addition to adenoviral vectors, the use of ncRNAs like nc886 could have broader applications across other gene therapy and delivery systems. For instance, lentiviral and adeno-associated viral (AAV) vectors could also benefit from similar strategies to reduce immune responses and improve transgene expression. This versatility highlights the potential of ncRNAs as a new class of tools for enhancing therapeutic gene delivery.^10^

The study raises several questions that warrant further exploration. Optimizing the levels of nc886 expression within the vector could enhance its efficacy while minimizing potential side effects. Future research should focus on fine-tuning nc886 expression to achieve the ideal balance between immune suppression and therapeutic benefit. Integrating nc886 with other approaches, such as immune checkpoint inhibitors or targeted delivery systems, could further enhance the efficacy of adenoviral vectors in specific contexts, such as cancer immunotherapy. Preclinical studies in animal models are a critical next step to evaluate the safety and efficacy of nc886-modified adenoviral vectors *in vivo*. Long-term studies will be particularly important to assess the durability of transgene expression and monitor for adverse effects. The potential of nc886 to enhance other therapeutic platforms, such as oncolytic viruses and RNA-based therapies, should be explored. Additionally, its use in diseases with strong immune components, such as autoimmune disorders, could open new avenues for treatment.

This study marks an important milestone in the optimization of adenoviral vectors for gene therapy. By leveraging the unique properties of nc886, the authors have addressed two critical challenges—immune activation and apoptosis—offering a promising solution for improving vector performance. While significant hurdles remain, including delivery efficiency and long-term safety, the insights gained from this research lay a strong foundation for future innovations.

As the field of gene therapy continues to evolve, the integration of ncRNAs like nc886 represents a powerful new tool for overcoming long-standing barriers.^11^ The authors’ work not only advances our understanding of the mechanisms underlying immune and apoptotic regulation but also provides a blueprint for developing safer and more effective gene delivery systems. With continued research and collaboration, this approach has the potential to transform the landscape of therapeutic gene delivery, offering hope for patients with previously untreatable conditions.

## Declaration of interests

The authors declare no competing interests.

## References

[1] Saruuldalai E., Lee H.H., Lee Y.S., Hong E.K., Ro S., Kim Y., Ahn T., Park J.L., Kim S.Y., Shin S.P. (2024). Adenovirus expressing nc886, an anti-interferon and anti-apoptotic non-coding RNA, is an improved gene delivery vector. Mol. Ther. Nucleic Acids.

[2] Fejer G., Freudenberg M., Greber U.F., Gyory I. (2011). Adenovirus-triggered innate signalling pathways. Eur. J. Microbiol. Immunol..

[3] Leen A.M., Christin A., Khalil M., Weiss H., Gee A.P., Brenner M.K., Heslop H.E., Rooney C.M., Bollard C.M. (2008). Identification of hexon-specific CD4 and CD8 T-cell epitopes for vaccine and immunotherapy. J. Virol..

[4] You Y., Cheng A.C., Wang M.S., Jia R.Y., Sun K.F., Yang Q., Wu Y., Zhu D., Chen S., Liu M.F. (2017). The suppression of apoptosis by alpha-herpesvirus. Cell Death Dis..

[5] Aurelian L. (2013). Oncolytic virotherapy: the questions and the promise. Oncolytic Virother..

[6] Wang J.H., Gessler D.J., Zhan W., Gallagher T.L., Gao G. (2024). Adeno-associated virus as a delivery vector for gene therapy of human diseases. Signal Transduct. Target. Ther..

[7] Davidson S., Maini M.K., Wack A. (2015). Disease-promoting effects of type I interferons in viral, bacterial, and coinfections. J. Interferon Cytokine Res..

[8] Tatsis N., Ertl H.C.J. (2004). Adenoviruses as vaccine vectors. Mol. Ther..

[9] Williams J.A. (2013). Vector Design for Improved DNA Vaccine Efficacy, Safety and Production. Vaccines (Basel).

[10] Mingozzi F., High K.A. (2013). Immune responses to AAV vectors: overcoming barriers to successful gene therapy. Blood.

[11] Uppaluri K.R., Challa H.J., Gaur A., Jain R., Krishna Vardhani K., Geddam A., Natya K., Aswini K., Palasamudram K., K S.M. (2023). Unlocking the potential of non-coding RNAs in cancer research and therapy. Transl. Oncol..

